# The influence of supervisor creative feedback environment on team creativity: The role of the ambidextrous learning and creative cognitive style

**DOI:** 10.3389/fpsyg.2022.1007947

**Published:** 2022-10-28

**Authors:** Shuwei Liu, Yawei Zhang, Yamei Liu, Linyan He, Yuchun Xiao

**Affiliations:** ^1^Zhejiang College of Security Technology, Wenzhou, China; ^2^School of Management, Zhejiang Shuren University, Hangzhou, China; ^3^Shenyang Institute of Engineering, Shenyang, China; ^4^School of Business Administration, Zhejiang Gongshang University, Hangzhou, China

**Keywords:** supervisor creative feedback environment, team creativity, ambidextrous learning, team creative cognitive style, exploratory learning

## Abstract

The survival and success of organizations increasingly depend on creativity. A Supervisor Creative Feedback Environment is of special value in enhancing team creativity, but few studies have explored the relationship between the supervisor creative feedback environment and creativity and how it affects creativity. Based on feedback intervention theory and triadic reciprocal determinism, this paper explores the process mechanism and boundary conditions of the supervisor creative feedback environment affecting team creativity from the perspectives of ambidextrous learning and team creative cognitive style. With 506 team members from 115 work teams in domestic enterprises as research samples, regression analysis was used to test the theoretical hypotheses. Feedback intervention, according to the feedback intervention theory, is a complicated process. There are various influencing factors, such as the feedback provider, means of feedback intervention, the content of the feedback information, situational factors, and the feedback recipients (Junwei, 2003). The leading creative feedback loop includes important feedback receiver's factors which are not mentioned above. Triadic reciprocal determinism holds that individual behavior is formed by the interaction and interconnection of individual, environment, and behavior. The two above-mentioned theories can explain why the leadership creative feedback environment can affect team creativity by influencing ambidextrous learning. The results also show that the feedback environment of supervisor creativity has positive effects on team creativity. Ambidextrous learning mediates the relation between supervisor creative feedback environments and team creativity. Team creative cognitive style has a positive moderating effect on the indirect relationship between a supervisor creative feedback environment and team creativity through ambidextrous learning. This study validates feedback intervention theory and triadic reciprocal determinism, expands the application of feedback environment factors in the research field of team creativity, provides a theoretical framework for the influence of the creative feedback environment on team creativity, and also provides theoretical support for managers to apply the management tool of a supervisor creative feedback environment to organizational context to improve team creativity. Based on the research results, this paper puts forward corresponding management suggestions from the aspect of creating a supervisor creative feedback environment, attaching importance to team ambidextrous learning, and making good use of creative cognitive style.

## Introduction

Innovation is the driving force for sustained economic growth (Zhi and Shudan, 2015), and innovation is critical to the success of companies especially in the context of green practices such as digital transition and carbon peaking and carbon neutrality; innovation is an important driving force to achieve these new changes, new requirements, and new goals. Creativity is the first step of innovation (Amabile, 1997). The survival and success of organizations increasingly depends on creativity (Gumusluoglu and Ilsev, 2009; Yoshida et al., 2014). The improvement of the innovation level of countries and enterprises depends on the improvement of the employees' creativity level. Creativity enables organizations to continuously innovate and maintain competitive advantages, which is the starting point of organizational innovation (Zhou, 2003). Management obsession with creativity is uncontrollable (Defillippi and Jones, 2004). While innovation is critical to an organization's success, many organizations may not be harnessing the creativity of all their employees (Taylor et al., 2020). As more and more enterprises adopt teams as a small, flexible, dynamic, and interactive organizational form to cope with rapid market changes (Richter et al., 2006), team creativity has gradually become one of the key factors for enterprises to achieve sustainable competitive advantages (Tjosvold et al., 2004). Therefore, how to enhance team creativity in the organization is crucial to an organization's innovation and success. There are many dependent variables to team creativity, and more and more people are interested in identifying environmental conditions that influence creativity (Shalley et al., 2004). Feedback is an important behavior correction and incentive strategy in organizations. High-quality feedback helps employees understand the progress of innovation and rectify issues and improve in a timely manner (Peng and Chiu, 2010), which aids in enhancing creativity. Independently, both formal feedback and informal feedback are insufficient. Therefore, it is necessary to create a feedback environment which is conducive to communication between the feedback parties (Zhenxing et al., 2019). The classic concept of a feedback environment (Steelman and Rutkowski, 2004) was proposed by Steelman et al. (2004). It goes beyond the feedback behavior itself, emphasizes the environmental factors related to feedback that can affect the effect of feedback behavior, highlights the usefulness of feedback, and solves the problem of focusing only on feedback behavior itself, which is insufficient to effectively improve organizational performance and employee self-development (Zhang et al., 2017). Feedback environment practices are more prone to intervention and can produce results in a shorter period of time (Whitaker et al., 2007). As a more comprehensive concept, a feedback environment is of great significance to creativity research and has become a hot topic in the field of creativity (Zhang et al., 2017). Past studies have focused on creative people and ignored the circumstances that facilitate creativity and thus pay less attention to the feedback environment.

The key to creativity is support. Creative feedback environments are different from traditional feedback environments. It pursues different quantities, speeds, and times of products and services, encourages employees to find better or unique solutions to problems in unknown areas, and gives employees time to think and explore (Shalley and Zhou, 2008). It is mainly composed of two dimensions: a supervisor creative feedback environment, and a colleague creative feedback environment. The supervisor creative feedback environment is a pointer to put forward novel and feasible ideas to the employee's work which are related to the products, services, processes; the supervisor gives reliable, high quality, and accurate feedback information in the proper way, supports feedback seeking, and offers time guarantee, tolerance, and targets expected situations for innovation (Zhenxing and Miaomiao, 2018). Colleagues are reluctant to provide feedback due to various considerations. Meanwhile, because supervisors often communicate with subordinates on behalf of the organization, they consequently have greater influence and power to reward and punish employees (Peng and Chiu, 2010), thereby playing a key role in enhancing employee creativity. Therefore, supervisors have a greater impact on the creative performance of the team (George and Zhou, 2007). The supervisor creative feedback environment has more supportive factors than an ordinary feedback environment. This will motivate employees to work in a more positive way, and it can provide employees with the information they need to promote the development of creative ideas. It may also increase the likelihood that feedback recipients will take action to change their behavior and improve creativity. It is a valuable perspective on improving team creativity.

There are few studies on Supervisor Creative Feedback Environments. In the literature, there are studies on the scale development of feedback environments (Steelman et al., 2004) and reviews (Zhang et al., 2017); contrastive studies of creative feedback environments (Gong and Li, 2018); studies on the relationship between feedback environments and creative performance (Gong and Zhang, 2017); studies on the relationship between colleagues feedback environments and employee creativity (Gong et al., 2019); studies on the influence of supervisor feedback environments on innovation performance (Zhang et al., 2017) and feedback environments on voice behavior (Zhang and Su, 2020); Supervisor Feedback Environment and Employee Creativity (Zhenxing et al., 2020), and so on. There is no research on the relationship between the Supervisor Creativity Feedback Environment and team creativity. Creative work is usually done in teams; therefore, to study the relationship between the supervisor creative feedback environment and creativity from the team level and explore the mediating and moderating factors will help managers to correctly understand the role of the Supervisor Creative Feedback Environment and find the tools to improve team creativity.

The formation of creativity requires the use of existing knowledge as well as new knowledge. Ambidextrous learning is an important way to improve creativity (Rothaermel and Deeds, 2004); it can enhance individual creativity (Rothaermel and Deeds, 2004). When teams undertake increasingly complex tasks, team members must complement each other with diversified knowledge and skills to achieve synergistic effects. Ambidextrous learning plays an important role in this process (Jiang et al., 2015) and is of great value to the study of creativity. Previous research on the relationship between ambidextrous learning and creativity are mostly from the individual level, but few from the team level. How to improve team creativity has become the focus of scholars' research and the tool which managers have been looking for. Therefore, it is of great value to study the impact of ambidextrous learning on creativity at the team level. Ambidextrous learning can take into account learning itself and the interaction between learning and the external environment (Fu-Qian et al., 2021). The supervisor creative feedback environment is likely to prompt the team members to learn by ambidextrous learning (exploratory learning and use of learning) to compensate for the lack of the knowledge and skills of creative work and to improve creativity according to valuable feedback information which is provided by the supervisor. Therefore, ambidextrous learning may be the mediating mechanism between the supervisor creative feedback environment and team creativity. However, in previous studies on the relationship between feedback environment and creativity, intrinsic motivation was considered as the main mechanism of environmental influence on creativity (Amabile, 1983, 1997). Environmental conditions affected the individual's creative performance by influencing an individual's intrinsic motivation (Zhou, 1998), and there have been a few studies on other mediating mechanisms.

Cognitive style also known as the way of cognitive, refers to the pattern of habitually solving problems, perceiving, thinking, and memorizing displayed by individuals in the cognitive process (Riding and Cheema, 1991). Different cognitive styles represent the individuals' preferences for information needs and information interaction (Epstein et al., 1996). Individual cognitive style can effectively predict creativity and is considered to be a decisive factor of individual creativity (Kim et al., 2012). Creative Cognitive style is conducive to the formation of creative thinking (Zhang H. et al., 2018). In the tripartite reciprocity model of social cognition theory, environment, cognition, and behavior influence each other, and people's behavior is jointly determined by environmental factors and their own cognition. Creativity is highest when employees have characteristics associated with creativity and perform complex tasks in a supportive and uncontrolled environment (Gerg and Cumming, 1996). As a thinking model and information processing model, creative cognitive style can build good working habits, working atmosphere, and team relationships (Epstein et al., 1996), and create supportive, harmonious, and happy working environments (Kirton, 1976), which is a characteristic related to creativity. The supervisor creative feedback environment is supportive and uncontrolled. Does creative cognitive style moderate the effect of the creative feedback environment on team creativity? Or does it moderate the mediating effect of ambidextrous learning on the supervisor creativity feedback environment and team creativity? Some previous studies have focused on the effect of creative cognitive style on creativity, but little attention has been paid to its effect on other variables, and there is no research on its effect on ambidextrous learning. The study is beneficial to expand the literature on creative cognitive style in theory and enhance the understanding of creative cognitive style in practice. Therefore, creative cognitive style can be correctly used in management.

With the increasing importance of team influence on creative work in organizations, answers to questions about how feedback environments influence creativity require a unique team-level perspective. Compared with the traditional feedback environment, the supervisor creative feedback environment is more valuable for research because of its pertinence, persistence, timeliness, and accuracy (Zhenxing and Miaomiao, 2018). The main concerns of this paper are as follows: Does the supervisor creative feedback environment influence team creativity? What is the mechanism of its influence? What role does creative cognitive style play in it? Does it affect the effect of the supervisor creative feedback environment on ambidextrous learning and on team creativity? The research on these concerns is of theoretical and practical significance, but still, there is little relevant research at present. Therefore, this study establishes a theoretical model based on feedback intervention theory and ambidextrous learning perspective, and reveals the influence of the supervisor creative feedback environment on creativity at the team level by integrating the supervisor creative feedback environment, ambidextrous learning, and creative cognitive style in the same research framework, exploring how it affects team creativity in stimulating team members' ambidextrous learning, and the moderating role of creative cognitive style. It mitigates the limitations of previous studies on the relationship between creative feedback environment and creativity and its mechanism, and is of great significance to the research on team creativity, feedback environment, ambidextrous learning, and creative cognitive style. It provides important theoretical support for managers to apply the supervisor creative feedback environment as a tool into management practice to improve team creativity. It also provides a theoretical basis for whether it is necessary to create a supervisor creative feedback environment in management practice, whether to pay attention to the cultivation of team ambidextrous learning ability, and whether it is necessary to consider the characteristics of creative cognitive style when selecting team members for creative work.

## Theoretical background and hypotheses

### Supervisor creative feedback environments and team creativity

In supervisor creative feedback environments, supervisors offer reliable, high-quality and accurate feedback on novel and feasible ideas related to products, services, and processes that are put forward by employees, support employees to seek feedback, and offer time guarantees, tolerance, and expectations (Zhenxing and Miaomiao, 2018). Organizational creativity theory and research emphasize the importance of creating a good working environment to enable employees' creativity (Amabile, 1997). Positive and stimulating work environments are consistently associated with creativity (Baer et al., 2003). Environmental factors can affect personal perceptions. When individuals perceive that their work is consistent with their interests and values, they may exhibit a higher level of creativity in the pursuit of goals (Zhang et al., 2017).

Feedback environments can provide employees with clear evaluation. The purpose and effect of feedback are obvious. In terms of the effectiveness of feedback, it provides employees with information to develop creative ideas (Whitaker et al., 2007). Certain dimensions of feedback environments may increase the likelihood that feedback recipients adjust and improve their creativity (Gong et al., 2019). The key to employee creativity is support (Gong et al., 2019). A highly supportive feedback environment can make employees feel appreciated and encourage them to work creatively (Sparr and Sonnentag, 2008) and with less uncertainty and ambiguity (Peng and Chiu, 2010), which helps improve their work performance and creativity (Whitaker and Levy, 2012; Young and Steelman, 2014). A supportive feedback environment makes individuals more cognitively flexible, pay more attention to complexity, and exhibit higher creativity (Zhang et al., 2017). Creative work requires thinking, time, and adventure (Gong and Li, 2018). Creative feedback environments support feedback seeking and innovation time, which is both supportive and motivating and can improve creative performance (Gong and Zhang, 2017).

Supervisors' support is a key feature of a creative work environment (Amabile et al., 2004); a supervisor creative feedback environment has more supporting factors than an ordinary feedback environment. It makes employees feel appreciated and motivates them to work in a more positive manner (Sparr and Sonnentag, 2008). Some scholars have verified the relation between supervisor feedback environments and employee creativity at the personal level and found a significant positive relation between them (Zhenxing et al., 2020). Research on team creativity originated from the practical needs of improving enterprise performance in the 1970s (Hongdan and Weiwei, 2018). Team creativity is the result of the interaction and joint action of individuals, teams, and contexts (Amabile, 1983) and is the ability of team members to generate novel and useful ideas through cooperation (Wang et al., 2016). The nature of team creativity is knowledge intensive and interdependent. At the team level, do supervisor creative feedback environments have a positive impact on team creativity? This study attempts to explore the influence of supervisor creative feedback environments on team creativity at the team level and predicts the following:

H1: Supervisory creative feedback environment has a significant positive effect on team creativity.

### Mediating effect of ambidextrous learning

Ambidextrous learning is the simultaneous pursuit of exploratory learning and exploitative learning (March, 1991). Exploitative learning includes refinement, efficiency, and practice. It is characterized by application and development, which can expand the breadth and depth of cognition (Amabile, 1997). Exploratory learning emphasizes moving beyond existing constraints to explore and create new knowledge (March, 1991). Binary learning is a dynamic task rather than a static matching (Raish et al., 2009). The interaction between learning itself and external environment can be considered (Fu-Qian et al., 2021), which promotes the introduction of creative ideas within the organization from different aspects to meet the requirements of team creativity. Ambidextrous learning is likely to mediate the relation between supervisor creative feedback environments and team creativity. The reasons are as follows:

First, context is an important factor in binary learning (Hirst and Zhou, 2009). According to social learning theory, individuals or groups seek resources from the environment to realize learning, and environmental factors play both modeling and supporting roles (Bandura, 1973). When employees perceive that they are supported by the external environment, their level of intrinsic motivation will increase, and they perform more beneficial actions for themselves or others (Yingya and Junchen, 2016). In a good feedback environment, employees know that their work is recognized and quickly receive responses (Steelman and Rutkowski, 2004); feedback interaction can directly affect task motivation. Success (confirmation of competence) leads to an increase in intrinsic motivation; higher levels of motivation may stimulate the learning of tasks and related subjects (Amabile, 1983). As an environment supporting innovation, a supervisor creative feedback environment may promote team learning by contributing to cognitive motivation and prosocial motivation.

Second, learning plays an important role in the team creation process (Hongdan and Weiwei, 2018). The formation of creativity requires both the use of existing knowledge and new knowledge. Binary learning is an important way to improve creativity (Rothaermel and Deeds, 2004). Exploratory learning can enrich the categories of team knowledge resources (Amabile, 1997). Through clear judgment and cognition of existing businesses and knowledge, enterprises should continuously accumulate, utilize, and optimize existing businesses and knowledge, realize the leap from quantitative change to qualitative change, and promote enterprises to explore new knowledge, new skills, and new fields (Qu and Zhang, 2021). Exploitative learning uses existing knowledge to select activities closely related to previous activities or that are highly repetitive. Its main features are “refining, integration, redevelopment, selection, production, implementation, and execution” (Politis, 2010). Exploitative learning can excavate the maximum value of existing experience and stimulate team members' willingness to innovate by promoting team members' ability to gain a sense of behavioral control (Zhang X. E. et al., 2018). The core of exploitative learning lies in improvement and extension (Xin-Mei et al., 2013). Both exploitative learning and exploratory learning can increase the knowledge reserve of enterprises and enhance their willingness and ability of cross-border innovation (Qu and Zhang, 2021). The complementary synergy of exploratory learning and exploitative learning can accelerate the formation and promotion of creativity (Xin-Mei et al., 2013). Balance between incremental innovation and breakthrough innovation can be achieved through the dynamic coordination of exploitative learning and exploratory learning, which is the key to building sustainable innovation capability, and the two types of learning need to achieve a balance rather than adoption or abandon (Gao et al., 2012). As two dimensions of binary learning, exploratory learning brings knowledge diversification and exploitative learning brings knowledge profoundness, which can exactly meet the requirements of team creativity (Xin-Mei et al., 2013).

Finally, the supervisor creative feedback environment may enhance team creativity by enhancing team members' motivation and ability to learn. The environment is the objective existence of the material entity, and the environment itself will not influence people's consciousness and behavior. It is only when people pay attention to the environment, that is, when the action is made real, the potential and value of the environment can be realized (Ling-zhi and Xiao-min, 2011). In a supervisor creative feedback environment, the quality and accuracy of feedback are high, and have tolerance and target expectations for employees' innovative work (Zhenxing and Miaomiao, 2018), which can enable team members to improve their logical thinking ability to explore and refine existing knowledge (Xin-Mei et al., 2013), and improve the efficiency of team knowledge integration and absorption (Atuahene-Gima and Murray, 2007). On the one hand, the close connection and exchange and sharing among team members (i.e., creative feedback environment) can reduce the cost of searching for new knowledge and technology; and on the other hand, it can improve the efficiency of team knowledge integration and absorption, and expand the breadth and depth of exploitative learning (Atuahene-Gima and Murray, 2007). In addition, according to the cognitive learning theory, the learning process is an active and selective information process made by individuals according to their own cognition of the external environment (Duanxu and Huijuan, 2013). The supervisor creative feedback environment supports innovation and is able to correctly confront the challenges brought by innovation and is willing to give employees time to learn creative skills (Gong and Zhang, 2017), which enables the team to have a deep understanding of the content and significance of tasks and can reduce the cost of searching for new knowledge and technology. It also enhances team members' experience and ability to cope with the challenge of exploring new knowledge and technology which is conducive to team exploratory learning. Based on this, this study proposes:

H2a: Exploratory learning mediates the relationship between supervisor creativity feedback environment and team creativity.
H2b: Exploitative learning mediates the relationship between supervisor creative feedback environment and team creativity.

### Moderating effect of team creative cognitive style

Cognitive style refers to the consistent differences in the way individuals seek, evaluate, organize, and process information (Gianluca and Balint, 2015). Kirton divides cognitive styles into Innovative Cognitive style and Adaptive Cognitive style (Kirton, 1976). Creative cognitive style provides a divergent and diversified thinking mode for the generation of breakthrough ideas (Kirton, 2003a,b). Individual cognitive style can effectively predict creativity and is considered to be a decisive factor of individual creativity (Kim et al., 2012). Factor theory suggests that creativity needs not only “will” but also “ability.” Creativity will reach the highest level when an individual has professional knowledge and skills, creation-related skills, and task motivation at the same time (Amabile and Pillemer, 2012). Employees with an adaptive style make incremental changes to “do better” within the existing structure. Employees with creativity cognitive styles take the current structure as part of the problem and make more radical changes by “doing things differently” (Martinsen, 2006). Individuals with creative cognitive style tend to choose high-risk and challenging jobs or tasks and prefer to pursue high risks and explore new things. Ambidextrous learning is the *ability* to form creativity while creative cognitive style is the *willingness* to form creativity.

Early studies on cognitive style pointed out that individual cognitive style not only determines the way of thinking, learning, decision-making, and coping, but also influences the establishment of interpersonal relationships and the interaction of peripheral information (Kirton, 1976). Cognitive style influences how individuals retrieve, organize, and interpret information in their environment and how interpretation of information is integrated into their mental models to guide subsequent actions (Hayes and Allinson, 1998). Team creative cognitive style is the aggregation of individual creative cognitive style. For teams with different creative cognitive styles, team members also have differences in perceiving learning motivation (Hirst and Zhou, 2009), and such perceived differences in learning motivation will have an impact on team learning ability and even the subsequent level of team creativity (Hongdan and Weiwei, 2018). In the tripartite reciprocity model of social cognition theory, environment, cognition, and behavior influence each other, and an individual's behavior is jointly determined by environmental factors and their own cognition. Environmental factors in organizations substantially affect the creative performance of employees, and the influence of these factors and practices may vary with the creative personality of employees (Amabile, 1997). According to the cognitive moderating mechanism in social cognitive theory, external feedback promotes individual cognitive moderation and indirectly affects individual behavior (Geng et al., 2020). A team's ambidextrous learning behavior is influenced by the team's creative cognitive style. Employees with lower creative cognitive style spend more time on exploitative learning, while employees with higher creative cognitive style not only pay attention to exploitative learning, but also have better exploratory learning ability. Based on the above analysis, team creative cognitive style may play a positive moderating role in the relationship between the supervisor creative feedback environment and exploratory learning and its relationship with exploitative learning. Based on this, the following hypotheses are proposed:

H3a: A team creative cognitive style positively moderates the relationship between a supervisor creative feedback environment and exploratory learning.
H3b: A team creative cognitive style positively moderates the relationship between a supervisor creative feedback environment and exploitative learning.

Different cognitive styles represent their information needs and interaction preferences (Epstein et al., 1996). Cognitive style influences employee behavioral decision-making and interpersonal interactions (Allinson et al., 2001). In an entrepreneurial team full of uncertainty, ambiguity, and complexity, cognitive style is not only related to the quantity and quality of social information delivered, but also affects team members' perception of enterprise dynamics and team atmosphere (Jia et al., 2021), which may have an important impact on team members' learning styles and engagement. Therefore, team creative cognitive style is an important contingency factor for organizational behavior. When a team has higher creative cognition, there is more exploratory learning or exploitative learning. When the team has lower creative cognitive style, there is less exploratory learning or exploitative learning. Exploratory learning and exploitative learning may be more effective in a higher team creative cognitive style. When individuals possess creative cognitive style and are accustomed to frequent external knowledge searches, they are more likely to produce breakthrough creativity (Zhang et al., 2021). On the one hand, the supervisor creative feedback environment indirectly affects team creativity through the mediating effects of exploratory learning or exploitative learning. On the other hand, team creative cognitive style positively moderates the relationship between supervisor creative feedback environment and exploratory learning and exploitative learning. Based on the analysis of these two aspects and the above analysis, this study further proposes that team creativity cognitive style also has a mediating effect on strengthening the exploratory learning in “supervisor creative feedback environment → exploratory learning → team creativity” and strengthening exploitative learning in “supervisor creative feedback environment → exploitative learning → team creativity,” which is actually moderated mediating effects. Based on the above analysis, the following hypotheses are proposed in this study:

H4a: A team creative cognitive style positively moderates the mediating effect of exploratory learning on the relationship between a supervisor creative feedback environment and team creativity, that is, the higher the team creative cognitive style is, the stronger the mediating effect of exploratory learning on the relationship between the supervisor creative feedback environment and team creativity.
H4b: A team creative cognitive style positively moderates the mediating effect of exploitative learning on the relationship between supervisor creative feedback environment and team creativity, namely, the higher the team creative cognitive style is, the stronger the mediating effect of exploitative learning on the relationship between the supervisor creative feedback environment and team creativity.

**Figure 1:**
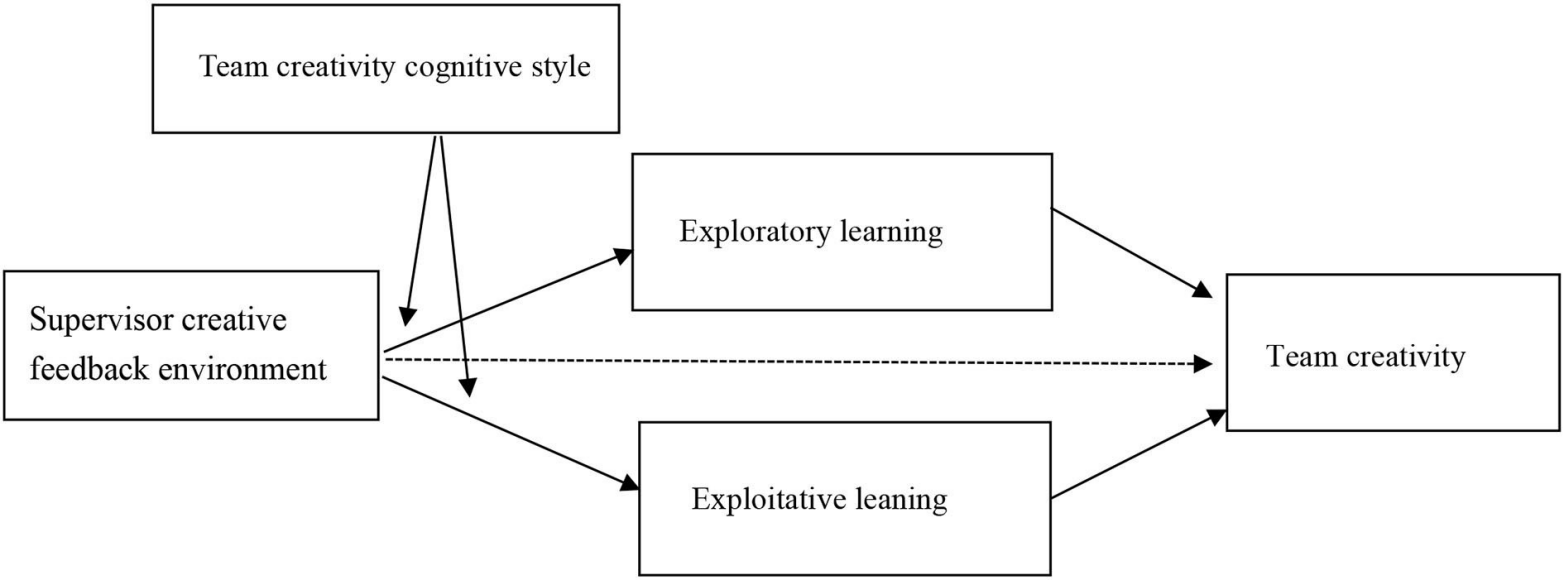
Research model.

Triad reciprocal determinism was proposed by American psychologist Bandura (2001, p. 35) based on Lewin's model. According to this theory, an individual's behavior is formed by the interconnection and interaction of three elements: the individual, the environment, and the behavior. According to the feedback intervention theory, feedback intervention is a complex process with various influencing factors, such as the provider of feedback valence, the feedback intervention method, the content of feedback information, situational factors, and the feedback receiver (Junwei, 2003). According to the two above-mentioned theories as well as the previous theoretical analysis and research hypotheses, this study constructs the theoretical model as shown in Figure 1.

## Research methodology

### Sample collection and data selection

Previous studies on feedback and team creativity mostly adopted experimental methods, and the teams studied were relatively weak in terms of power distribution and temporal stability. In this study, the research objects are teams with more temporal stability, because their previous history may affect their response to feedback. The research data were collected from eight enterprises in Guangdong, Zhejiang, Jilin, and Hebei provinces, covering sectors that require creativity in jobs in the industry of finance, energy, manufacturing, healthcare, and education.

First, the team supervisor enabled the collection of creation data using the supervisor-member matching survey method. The survey was performed in two parts. First, the evaluation of team creativity was completed by the team supervisor, which matches the evaluation of the creative feedback environment of the subordinates, binary learning, and team creativity cognitive style. In addition, demographic information was provided. In this study, 562 questionnaires were distributed to 125 teams. After matching and integrating the collected questionnaires, 506 valid questionnaires were obtained, including 115 from supervisors and 391 from employees. Among the 302 employees, 216 were male, which accounted for 55.2% of the sample. There were 253 employees over the age of 30, accounting for 64.8% of the sample. In terms of education, 287 people (73.5%) had a junior college or higher education. In terms of the working years of the team, 129 people had worked with the team for more than 5 years, accounting for 32.9% of the sample. The average size of the 115 teams was 6.51 people. For team level, demographic variables such as team creativity and supervisor's age, gender, and tenure were collected; for individuals, variables such as supervisor's creative feedback environment, ambidextrous learning, and team creative cognitive style were collected.

### Variables and measures

All scales in this study were taken from mature scales developed domestically and internationally. The meaning of the items in each variable scale were checked for agreement with the meaning represented by each variable in this research model and the overall content to be studied by the model and found to be basically the same. Therefore, the items in the corresponding scales of the following variables are used as the contents of the questionnaire. Except for the control variables, all variables were evaluated using a 7-point Likert scale, where 1 means “completely inconsistent” and 7 means “completely consistent.” A higher value indicates a higher degree of recognition of the statement.

#### Supervisor creative feedback environment

The supervisor creative feedback environment scale developed by Gong and Zhang (2017) was adopted in this study. It contains 16 items and mainly includes the credibility of the feedback source, quality, accuracy, mode availability, support feedback seeking, time guarantee of innovation, tolerance, and innovation goals of seven subsystem dimensions (Gong and Li, 2018). Typical measurement items include “I have many opportunities to work and interact with supervisors” and “Supervisors give me enough time to explore different viewpoints and ideas.”

#### Team creativity

The scale of Shin and Zhou (2007) was adopted; it consists of 4 items. Typical items include “Our team always puts forward good new ideas” and “Our team comes up with new ideas that are important to the business.” This variable is evaluated directly by the supervisor and the score is for the team level.

#### Ambidextrous learning

The scale of Kostopoulos and Bozionelos (2011) was used. It includes 5 items measuring exploitative learning. Typical items include “team members use their past knowledge to complete work” and “team members mainly use their existing knowledge and skills to carry out work.” Exploratory learning is measured by five items, such as “Team members try to complete tasks in new and creative ways” and “Team members develop many new skills in their work.”

#### Team creativity cognitive style

The scale of Miron et al. (2004) was adopted; it includes 4 items. Typical items are “I like work that allows me to exert my creative thinking” and “I like to do things in original ways.”

#### Control variables

Since the factors that affect team creativity may include demographic variables (Bernerth and Aguinis, 2016), average team tenure may affect team creativity (Shin and Zhou, 2007). Searching the literature for similar studies revealed that most control for the age, gender, and tenure of the supervisors (Vandenberghe et al., 2019), so this study also controls for these variables.

## Results analysis

In this study, MPLUS 7.0 and SPSS 23.0 software were used to analyze the data.

### Common method bias

In order to test the deviation of data, we used confirmatory factor analysis to compare the fit of different models. These models include five factors (five factors are: supervisor creative feedback environment, exploratory learning, exploitative learning, team creative cognitive style, and team creativity; the fitting index was *X*^2^ = 795.081; *Df* = 517, *x*^2^*/df* = 1.538; CFI = 0.967; TLI = 0.964; RMSEA = 0.037); four-factor model (items of supervisor creative feedback environment and exploratory learning as one factor, items of other variables as the remaining three factors), fitting index *x*^2^ = 1,785.819; *Df* = 521, *x*^2^*/df* = 3.428; CFI = 0.851; TLI = 0.839; RMSEA = 0.079); three-factor model (the items of supervisor creative feedback environment, exploratory learning, and exploitative learning are taken as one factor, the items of other variables are the remaining two factors, and the fitting index is *x*^2^ = 2,981.894; *Df* = 524, *x*^2^*/df* = 5.691; CFI = 0.710; TLI = 0.689; RMSEA = 0.110); two-factor model (supervisor creative feedback environment, exploratory learning, exploitative learning, team creative cognitive style as one factor, other variables as the remaining one factor. The fitting indexes were *x*^2^ = 3,884.736; *Df* = 526, *x*^2^*/df* = 7.385; CFI = 0.603; TLI = 0.577; RMSEA = 0.128); one-factor model (all variables are taken as one factor, and the fitting index is *x*^2^ = 4,734.652; *Df* = 527, *x*^2^*/df* = 8.984; CFI = 0.503; TLI = 0.471; RMSEA = 0.143).

It can be seen from the above results that the fitting effect of the five-factor model is better than other nested models, indicating that there is good discriminant validity among variables, and that the measurement of this study is reliable, and the structural attributes are good, which is suitable for subsequent data analysis. In addition, we compared the common method latent factor (CMV) model on the basis of five factors (the fitting index is *x*^2^ = 730.767; *Df* = 493, *x*^2^*/df* = 1.482; CFI = 0.972; TLI = 0.968; RMSEA = 0.035), and it is found that after the addition of common method potential factor (CMV), ΔCFI = 0.005, Δ, TFI = 0.004, ΔRMSEA = −0.002, the improvement of fit degree is weak, indicating that there is no serious common method deviation in this study.

### Reliability and validity test

First, a confirmatory factor analysis (CFA) was performed on the variables in the model with MPLUS 7.0. The results are shown in Table 1. The load of each variable was 0.643–0.899, and most were above 0.70. The composite reliability (CR) of the supervisor creative feedback environment was 0.948, that of exploratory learning was 0.890, that of exploitative learning was 0.920, that of team creativity was 0.902, and that of team creativity cognitive style was 0.891. All values were >0.70; the average extraction variance (AVE) of the supervisor creative feedback environment was 0.535, that of exploratory learning was 0.618, that of exploitative learning was 0.698, that of team creativity was 0.698, and that of team creativity cognitive style was 0.674. The AVEs of all variables were >0.50, indicating that the scale has good internal consistency, reliability, and aggregation validity.

**Table 1:** Construct factor loading and reliability.

**Dim**	**Item**	**Parameters of significant test**				**Item reliability**	**Composite reliability**	**Convergence validity**
		**Estimate**	**S.E**.	**Est./S.E**.	***P*-Value**	**R-square**	**CR**	**AVE**
LCE	LCE1	0.701	0.027	25.793	***	0.492	0.948	0.535
	LCE2	0.741	0.024	30.583	***	0.550		
	LCE3	0.741	0.024	30.517	***	0.549		
	LCE4	0.756	0.023	32.674	***	0.572		
	LCE5	0.751	0.024	31.831	***	0.563		
	LCE6	0.735	0.025	29.717	***	0.540		
	LCE7	0.757	0.023	32.746	***	0.573		
	LCE8	0.745	0.024	30.977	***	0.554		
	LCE9	0.679	0.029	23.645	***	0.461		
	LCE10	0.734	0.025	29.632	***	0.539		
	LCE11	0.751	0.024	31.960	***	0.565		
	LCE12	0.719	0.026	27.746	***	0.517		
	LCE13	0.709	0.027	26.606	***	0.502		
	LCE14	0.717	0.026	27.524	***	0.514		
	LCE15	0.695	0.028	25.178	***	0.483		
	LCE16	0.763	0.023	33.615	***	0.581		
EL	EL1	0.707	0.029	24.383	***	0.499	0.890	0.618
	EL2	0.792	0.023	34.417	***	0.628		
	EL3	0.860	0.018	47.633	***	0.739		
	EL4	0.813	0.021	38.103	***	0.662		
	EL5	0.750	0.026	29.186	***	0.563		
LL	LL1	0.805	0.021	38.985	***	0.648	0.920	0.698
	LL2	0.848	0.017	48.804	***	0.719		
	LL3	0.861	0.016	53.150	***	0.741		
	LL4	0.865	0.016	54.081	***	0.747		
	LL5	0.797	0.021	37.617	***	0.636		
CRS	CRS1	0.855	0.017	48.884	***	0.731	0.891	0.674
	CRS2	0.862	0.017	50.835	***	0.743		
	CRS3	0.899	0.015	60.591	***	0.809		
	CRS4	0.643	0.032	19.896	***	0.413		
ICE	ICE1	0.842	0.019	44.589	***	0.709	0.902	0.698
	ICE2	0.811	0.021	38.349	***	0.658		
	ICE3	0.874	0.017	52.178	***	0.764		
	ICE4	0.812	0.021	38.779	***	0.659		

LCE, supervisor creative feedback environment; EL, exploratory learning; LL, exploitative learning; CRS, team creativity cognitive style; ICE, team creativity. ***P < 0.001.

### Data aggregation test

This research was conducted at the team level, and individual measurements of team members were first aggregated to the team level. For the core variables, team creativity was directly evaluated by the supervisors and scored at the team level with no need for aggregation. Other variables, such as the supervisor creative feedback environment, exploratory learning, exploitative learning, and team creativity cognitive style, were aggregated from the individual level to team-level data. Based on the results of the polymerization analysis, supervisor creative feedback environment [ICC (1) = 0.362, ICC (2) = 0.669], exploratory learning [ICC (1) = 0.404, ICC (2) = 0.700], exploitative learning [ICC (1) = 0.381, ICC (2) = 0.687] and team creative cognitive style [ICC (1) = 0.368, ICC (2) = 0.680]. The ICC1 values of these four variables were all between 0.10 and 0.50, indicating that the variables had appropriate inter-group differences. The ICC2 of one of these four variables is >0.70, and the other three are close to 0.70, indicating that these four variables have good inter-group reliability. Meanwhile, the mean Rwg values of supervisor creative feedback environment, exploratory learning, exploitative learning, and team creative cognitive style are 0.908, 0.972, 0.911, and 0.880 respectively, which are all higher than 0.70 and relatively high. In general, Rwg, ICC1, and ICC2 values of these four variables are ideal and suitable for aggregation to the team level.

### Variables statistical descriptive and correlation coefficient analysis

Table 2 shows the analysis at the team level, including the average and standard deviation of each major variable and the correlation coefficient among the variables. The correlation coefficients of the supervisor creative feedback environment, exploratory learning, exploitative learning, and team creativity were 0.467 (*P* < 0.01), 0.458 (*P* < 0.01), and 0.469 (*P* < 0.01), respectively, indicating that the supervisor creative feedback environment is positively correlated with exploratory learning, exploitative learning, and team creativity. The correlation coefficient between exploratory learning and team creativity was 0.639 (*P* < 0.01), indicating a positive correlation between exploratory learning and team creativity. The correlation coefficient between exploitative learning and team creativity was 0.587 (*P* < 0.01), indicating that there was also a positive correlation between exploitative learning and team creativity.

**Table 2:** Descriptive analysis and correlation coefficient of variables.

**Variables**	***Mean***	***SD***	**1**	**2**	**3**	**4**	**5**	**6**	**7**
1. Age of team supervisors	3.080	1.334	1						
2. Gender of team supervisors	0.367	0.484	−0.076	1					
3. Tenure of team supervisors in months	50.450	49.194	0.301**	0.035	1				
4. Supervisor creative feedback environment	4.760	0.900	0.065	−0.148	0.191*	1			
5. Exploratory learning	5.314	0.880	0.245*	−0.202*	0.191*	0.467**	1		
6. Exploitative learning	5.241	0.878	0.162	−0.157	0.124	0.458**	0.649**	1	
7. Creativity cognitive style	4.582	1.012	0.057	−0.062	−0.026	0.304**	0.210*	0.232*	1
8. Team creativity	4.863	0.932	0.130	−0.072	0.073	0.469**	0.639**	0.587**	0.153

This table is a team-level analysis; continuous variables of supervisor age coded in subsections as 1–6 (1. under 30 years old; 2. 30–35 years old, 3. 36–40 years old, 4. 41–45 years old, 5. 46–50 years old, 6. 50 years old or above). The values in the table are standardized regression coefficients,

^*^P < 0.05 and

^**^P < 0.01.

### Hypothesis testing

#### Main effect test

In this paper, hierarchical linear regression analysis was used to test the hypothesis, and the regression analysis results are shown in Table 3. Model 1 showed that, after controlling for the age, gender, and tenure of team supervisors, there was a positive correlation between the supervisor creative feedback environment and team creativity (*b* = 0.472, *SE* = 0.089, *t* = 5.330,), and H1 was verified.

**Table 3:** Summary regression table of moderating mediation model.

**Variables**	**Team creativity**			**Exploratory learning**			**Exploitative learning**			**Team creativity**		
	**M1**			**M2**			**M3**			**M4**		
	***b***	***SE***	***t***	***b***	***SE***	***t***	***b***	***SE***	***t***	***b***	***SE***	***t***
**(Constant)**												
**Control variables**		
Team supervisee age	0.116	0.090	1.281	0.121	0.085	1.422	0.035	0.085	0.417	0.004	0.076	0.054
Team supervisee gender	0.008	0.087	0.096	−0.049	0.083	−0.592	0.016	0.083	0.190	0.082	0.073	1.134
Team supervisee tenure	−0.052	0.092	−0.565	0.055	0.085	0.642	0.003	0.085	0.039	−0.077	0.076	−1.021
**Independent variable**		
Supervisor creative feedback environment	0.472	0.089	5.330***	0.525	0.092	5.682***	0.563	0.092	6.132***	0.184	0.083	2.210*
**Mediating variables**		
Exploratory learning										0.420	0.099	4.265***
Exploitative leaning										0.252	0.096	2.635**
**Moderating variables**	
Team creativity cognitive style				0.015	0.0850	0.171	0.025	0.084	0.295			
Supervisor creative feedback environment* team creativity cognitive style				0.290	0.082	3.550***	0.372	0.081	4.582***			
*R^2^*				0.365			0.372			0.492		
**Indirect effects**				**Mediating variables**			效应	**SE**	**Boot 95% CI**
				Exploratory learning			0.221	0.097	[0.0595, 0.4434]
				Exploitative learning			0.142	0.079	[0.0212, 0.3371]

^*^P < 0.05,

^**^P < 0.01, and

^***^P < 0.001.

#### Mediating effects

In this study, a self-repeating sampling method was used to select model 7. The independent variable was the supervisor creative feedback environment, and the dependent variables were exploratory learning, exploitative learning, and team creativity. Repeated sampling for 5,000 times was conducted by process software, and the results are shown in Table 3. From M2 in Table 3, after controlling for age, gender, and tenure of supervisors, the supervisor creative feedback environment is positively correlated with exploratory learning (*b* = 0.525, *SE* = 0.092, *T* = 5.682). According to M3, supervisor creative feedback environment is positively correlated with exploitative learning (*b* = 0.563, *SE* = 0.092, *T* = 6.132). M4 shows that exploratory learning is positively correlated with team creativity (*b* = 0.420, *SE* = 0.099, *T* = 4.265), and exploitative learning is positively correlated with team creativity (*b* = 0.252, *SE* = 0.096, *T* = 2.635). After mediating variables were included, there was still a positive correlation between the supervisor creativity feedback environment and team creativity (*b* = 0.184, *SE* = 0.083, *T* = 2.210). It can be concluded that exploratory learning and exploitative learning play a partially mediating role in the supervisor creative feedback environment and team creativity. The mediating effect of exploratory learning was *b* = 0.221, *SE* = 0.097, Boot 95% CI = [0.0595, 0.4434]. The mediating effect of exploitative learning is *b* = 0.142, *SE* = 0.079, Boot 95% CI = [0.0212, 0.3371]. Hypothesis 2a and 2b are verified.

#### Moderating effects

According to M2 in Table 3, the interaction items of the supervisor creative feedback environment and creative cognitive style of team are positively correlated with exploratory learning (*b* = 0.290, *SE* = 0.082, *T* = 3.550). According to M3, the interaction item between supervisor creative feedback environment and team creative cognitive style has a significant positive impact on exploitative learning (*b* = 0.372, *SE* = 0.081, *T* = 4.582). Team creative cognitive style plays a significant positive moderating role in the relationship between supervisor creative feedback environment and exploratory learning and exploitative learning, respectively, concluding that H3a and H3b are supported.

**Figure 2:**
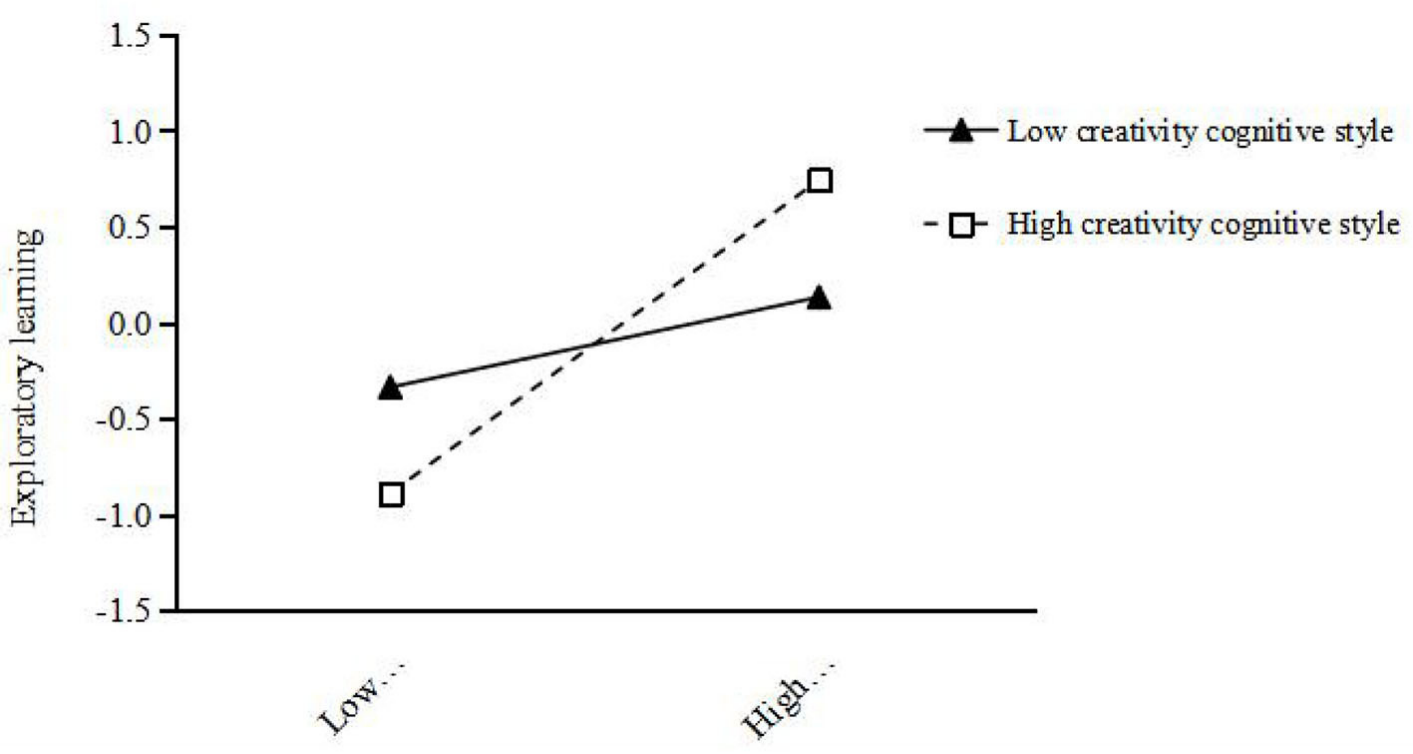
Regulatory effect of creativity cognitive style on the indirect relation between exploratory learning and supervisor creative feedback environment.

**Figure 3:**
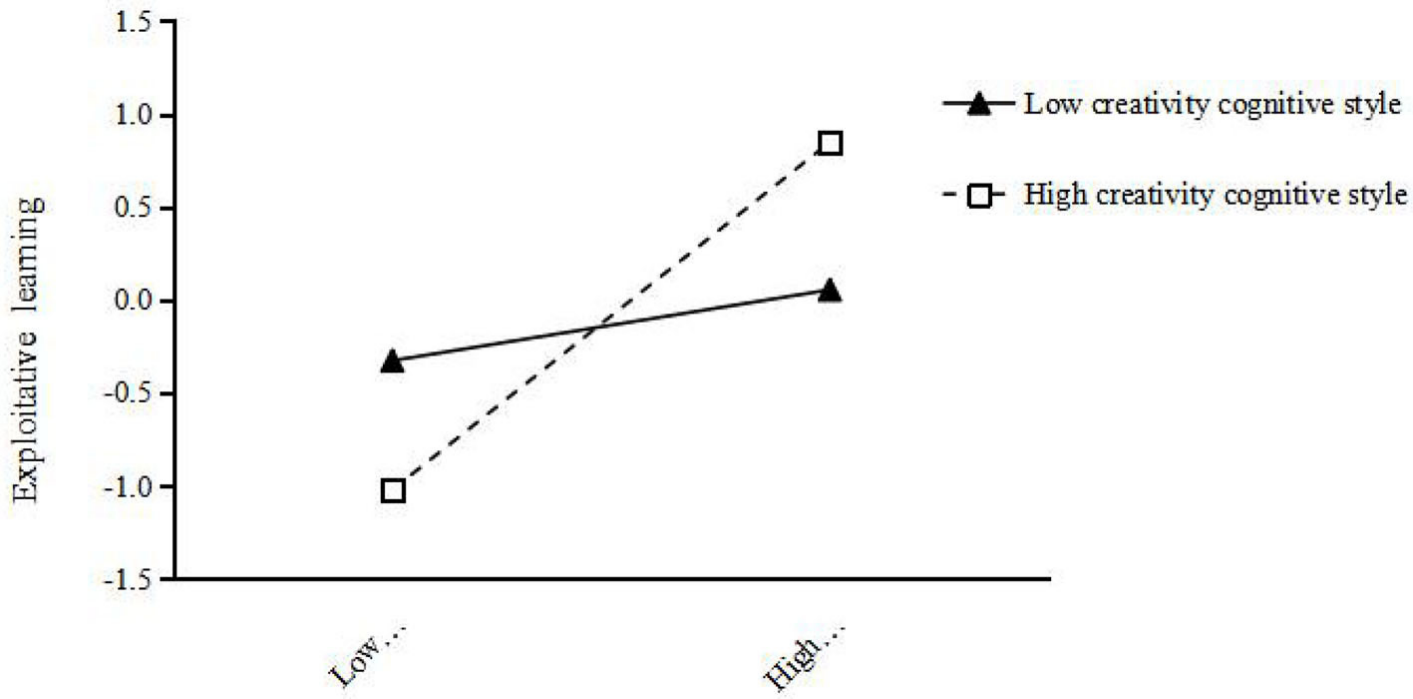
Regulatory effect of creativity cognitive style on the indirect relation between exploitative learning and supervisor creative feedback environment.

For further validation of team creativity cognitive style adjustment, team creativity cognitive styles can be divided into high and low team creativity cognitive style groups, and the two cognitive styles were drawn under the level of leading creative team feedback environment's impact on exploratory study and use of the learning effect diagram (see Figures 2, 3). As can be seen from Figures 2, 3, regardless of the level of team creative cognitive style, the supervisor creative feedback environment has a positive impact on exploratory learning and exploitative learning. However, in the case of high-team creative cognitive style, the slope of the corresponding line relative to the horizontal axis is larger, that is, the slope of the corresponding line is larger, indicating that the supervisor creative feedback environment has a stronger positive effect on exploratory learning and exploitative learning in the case of high-team creative cognitive style.

#### Moderated mediating effects

Bootstrap method was used to further distinguish the mediating effects of exploratory learning and exploitative learning under different conditions. The results are shown in Table 4. As can be seen from Table 4, when the level of team creative cognitive style is relatively high, the mediating effect value of exploratory learning = 0.343, *SE* = 0.145, 95% confidence interval [0.102, 0.682], the mediating effect value of exploitative learning = 0.235, *SE* = 0.126, 95% confidence interval [0.035, 0.538]; When the level of team creative cognitive style is relatively low, the mediating effect value of exploratory learning = 0.099, *SE* = 0.060, 95% confidence interval [0.009, 0.256]; the mediating effect value of exploitative learning = 0.048, *SE* = 0.044, 95% confidence interval [−0.008, 0.178]. The cognitive style of team creativity has a significant moderating effect on the mediating mechanism of “supervisor creative feedback environment → exploratory learning → team creativity” and “supervisor creative feedback environment → exploitative learning → team creativity.” Hypothesis H4a and hypothesis H4b are verified.

**Table 4:** Conditional indirect effects.

**Supervisor creative feedback environment**→**Exploratory learning**→**Team creativity**				
**Moderator variables**	**Level**	**Effect value**	**SE**	**Boot 95% CI**
Team creativity cognitive style	High	0.343	0.145	[0.102, 0.682]
	Medium	0.221	0.097	[0.060, 0.443]
	Low	0.099	0.060	[0.009, 0.256]
Team creativity cognitive style	High	0.235	0.126	[0.035, 0.538]
	Medium	0.142	0.079	[0.021, 0.337]
	Low	0.048	0.044	[−0.008, 0.178]

The “high/low” level of the moderator is one standard deviation above/below the average.

## Discussion

Supervisor Creative Feedback Environment is particularly valuable in enhancing creativity. Based on feedback intervention theory and triadic reciprocal determinism, this paper discusses the process mechanism and boundary conditions of the influence of the supervisor creative feedback environment on team creativity from the perspectives of ambidextrous learning and team creative cognitive style. The results show that the supervisor creative feedback environment positively affects team creativity; ambidextrous learning mediates the relationship between the supervisor creative feedback environment and team creativity; creative cognitive style has a positive moderating effect on the indirect relationship between the supervisor creative feedback environment and team creativity through ambidextrous learning. The research extends the application of feedback environment factors in the field of team creativity; it provides a theoretical framework for the influence of the supervisor creative feedback environment on team creativity, and provides theoretical support for managers to apply the management tool of the supervisor creative feedback environment to organizational situations to improve team creativity. Although the research adopted the method of matching leaders and members, which can reduce some common method bias problems, due to the questionnaire survey method and the limitations of sample size and geographical conditions, the universality of the research conclusions still needs to be further verified. But the conclusion has strong theoretical and practical significance.

### Theoretical implications

Based on the feedback intervention theory, this study explores the influence of the supervisor creative feedback environment on creativity from the team level, as well as the mediating and moderating effects of ambidextrous learning and team creative cognitive style on the above relationship, and extends existing research in the following aspects.

First, in view of the conceptual characteristics of team creativity, this study verifies whether the supervisor creative feedback environment has an impact on team creativity and the mechanism of this impact. The research on the relationship between supervisor creative feedback environments and creativity is extended to the team level. In the past, little attention has been given to the relation between feedback environments and creativity with only a few studies having been conducted. There are meanings and dynamics in teams that cannot be explained or covered by individual characteristics. At the team level, this study verifies the impact of supervisor creative feedback environments on creativity and the indirect impact of the interaction between supervisor creative feedback environments and team creativity cognitive style on creativity. The results provide new ideas for studying team creativity and expand the research on feedback intervention theory at the team level.

Second, the study focuses on the learning mechanism that affects team creativity and reveals the complex mechanism of supervisor creative feedback environments on team creativity from the perspective of ambidextrous learning, which conforms to the trend of ambidextrous learning research and promotes the theoretical transformation of the mechanism of feedback environment on team creativity. Previous studies mostly studied the influence mechanism of environment on team creativity from the perspective of intrinsic motivation. This study explored how the supervisor creative feedback environment affects team creativity from the perspective of ambidextrous learning, which opens up a new direction for the study of feedback environment and team creativity. The results of this study support the view that ambidextrous learning is an important mechanism to explain the relationship between supervisor creative feedback environments and team creativity. This finding lays a foundation for examining the influence mechanism of different types of feedback environments on team creativity in the future, and opens up a new perspective for the study of ambidextrous learning.

Third, the study takes creative cognitive style as a situational factor influencing team creativity and examines how the interaction between creative cognitive style and supervisor creative feedback environments affect team creativity through team ambidextrous learning and how it directly affects team creativity. This study answers the question “why does the same supervisor creative feedback environment lead to different team creativity.” Previous studies on creativity pay little attention to how team characteristics affect team creativity and lack systematic research on the importance of creative cognitive style in the process of influencing team creativity. In this study, we found that the influence of supervisor creative feedback environments on the intervention of team creativity and on ambidextrous learning depends on the team creative cognitive style, which extends the boundary conditions of team creativity research and contributes to the literature on the relationship between cognitive style and ambidextrous learning and between it and team creativity.

### Managerial implications

The practical contributions of this study are mainly as follows:

First, it provides a new perspective for managers to improve team creativity. The research proves that a supervisor creative feedback environment is an effective method for improving team creativity and provides valuable suggestions for managers to improve team creativity. In the process of innovation, more teamwork is needed, and team creativity is often generated in co-creation processes. Managers should try to build a creative feedback environment to properly provide credible, high-quality, and accurate feedback on the novel and feasible ideas of employees in their work, support feedback seeking, and provide innovation time guarantees, tolerances, and expectations. Organizations should strengthen the cultivation of supervisors' ability to construct creative feedback environments, give full play to the role of supervisor creative feedback environments in enhancing team creativity, help managers break the innovation paradox, and benefit from focusing on creating supervisor creative feedback environments that support creativity. In this feedback environment, the team can generate more creativity through ambidextrous learning and collaboration among team members.

Second, the conclusion that team ambidextrous learning significantly affects team creativity is of positive significance to managers' understanding that how to improve team creativity through learning management in management practices. The results of this study clarify the impact of ambidextrous learning on team creativity and compel managers to pay more attention to the unique value of ambidextrous learning. Both exploratory learning and exploitative learning are beneficial to improve team creativity. Therefore, managers should not only pay attention to exploratory learning, which is the basis of creativity, but also pay attention to exploitative learning, which can enhance creativity by developing the applied value of knowledge. Managers can improve the team's ambidextrous learning ability by building a creative feedback environment, extending the creative feedback environment within the company, and also by hiring external experts to give feedback on the team's current working status to bring new knowledge and new perspectives to enable team members to work regularly in a learning way that is conducive to team creativity.

Third, the discovery of the moderating effect of creative cognitive style inspires managers that creative cognitive style is the boundary condition that affects the effect of supervisor creative feedback environments on ambidextrous learning and team creativity. In order to enhance the effect of supervisor creative feedback environments on ambidextrous learning and team creativity, managers should pay attention to team creative cognitive style. When selecting team members who need to complete creative work, the characteristics of their creative cognitive style should be considered, and more members with high creative cognitive style should be selected or their creative cognitive style should be improved through internal training. If the enterprise cannot change the creative cognitive style of team members due to limited conditions, managers should exert more efforts to enhance the team's ambidextrous learning ability by enhancing the supervisor creative feedback environment, so as to improve team creativity.

### Limitations and future research

Restricted by the research conditions, this study has some limitations. First, there was an insufficient sample size and small regional scope. The sample number of this study is only 115 work teams, 506 team members, the regions involved total 8 enterprises in 4 provinces, and the industries involved are only five industries that are closely related to creativity. The reliability and validity of the findings might have been better with a larger sample size. Future studies should expand the sample size and sample industries to make the data sources more diverse. Second, this study adopted a questionnaire investigation method. Self-evaluation, other evaluation, and a pairing of supervisors and team members were used to answer the questionnaires. However, due to the limitation of objective conditions, the data, which were measured at only one time point, cannot reflect vertical causal relations between the variables. Multiple time points must be longitudinally measured in the future. In addition, in terms of team size, some teams are small. For the sake of data authenticity, these data are retained, but the reliability of data may be reduced. Third, this study explores the influence of supervisor creative feedback environments on team creativity from the perspective of the learning mechanism, although it expands the understanding of the mechanism by which environmental factors influence creativity, this study remains insufficiently comprehensive. In the future, the influence of mediating mechanisms such as emotion and relation coordination should be probed. In addition to supervisor creative feedback environments, colleague creative feedback environments and mentor feedback environments may also influence team creativity. Future research should focus on additional factors that may influence team creativity in the field of feedback environments to provide a more theoretical basis for improving team creativity from the perspective of feedback environments. Green practices and digital technologies are areas where creativity is in high demand; future directions related to the intersection of green practices and digital technologies may be beneficial.

## Conclusion

This study empirically examines the mechanism of the supervisor creative feedback environment on team creativity, verifies most hypotheses, and draws the following important conclusions:

The supervisor creative feedback environment positively affects team creativity; it has a positive influence on both dimensions of ambidextrous learning (exploitative learning and exploratory learning). Exploratory learning plays a mediating role between the supervisor creative feedback environment and team creativity; exploitative learning also mediates the relationship between the supervisor creative feedback environment and team creativity. Team creative cognitive style positively moderated the relationship between the supervisor creative feedback environment and exploratory learning and the relationship between supervisor creative feedback environment and exploitative learning. Team creative cognitive style positively moderated the mediating effect of exploratory learning on the supervisor creative feedback environment and team creativity, namely, the higher team creative cognitive style, the stronger the mediating effect of exploratory learning on the supervisor creative feedback environment and team creativity. Team creative cognitive style positively moderated the mediating effect of exploitive learning on the relationship between the supervisor creative feedback environment and team creativity, namely, the higher team creative cognitive style, the stronger the mediating effect of exploitive learning on the relationship between supervisor creative feedback environment and team creativity.

## Data availability statement

The raw data supporting the conclusions of this article will be made available by the authors, without undue reservation.

## Author contributions

SL was responsible for the theoretical model construction, questionnaire design, data analysis, and paper writing. YZ was responsible for data collection and entry, responsible for part of the data statistical analysis, and the contact and revision of the paper. YL was responsible for the text revision and English language translation. LH was responsible for proofreading the article. All authors contributed to the article and approved the submitted version.

## Funding

This research was supported by a research grant from the National Science Foundation of China (Grant No. 72074195).

## Conflict of interest

The authors declare that the research was conducted in the absence of any commercial or financial relationships that could be construed as a potential conflict of interest.

## Publisher's note

All claims expressed in this article are solely those of the authors and do not necessarily represent those of their affiliated organizations, or those of the publisher, the editors and the reviewers. Any product that may be evaluated in this article, or claim that may be made by its manufacturer, is not guaranteed or endorsed by the publisher.
